# The Nursing Supplement

**Published:** 1890-03-22

**Authors:** 


Being the Extra Nursing Supplement of "The Hospital" Newspaper.

All Contributions for this Supplement should be addressed to the Editor, The Hospital, 140, Strand, London, W.C., and should have the word "Nursing" plainly written in left-hand top corner of the envelope.

### En Passant.

A HINT TO MATRONS.—We notice that the reports issued by the various institutions frequently omit to give the Christian names of the matrons. We call attention to the point because it places the matrons at a disadvantage on account of the difficulty of identifying her where, as in a recent case, there are six Miss Smiths, five Miss Browns, and three Miss Robinsons among the applicants for an appointment. Men emphasize their identity by selecting a distinctive Christian name, which they always give in full. Thus we have the Trumpeter-Smiths, the Farrier-Smiths, and the Smith-Browns, and so on. Now that women are coming more and more to the front as workers in this busy world we would suggest that they should follow the men's example, at any rate so far as hospital matrons are concerned, and that they should request the secretary to always give their first Christian names in full in the reports.

BURTON NURSING INSTITUTION.—At the fifth annual meeting of this institution, the chairman (Mr. Grinling) said that there were 439 cases treated during the year, and about 11,000 visits paid. There had been a great deal of *bona fide* work done. There were four nurses engaged for this work, and every nurse cost £50 to £60 a year. At present they managed to pay their way. They left off with a balance of £18 in hand, as far as district nursing was concerned, but they were £6 to the bad, as they commenced the year with a balance of £24. There were two or three wants which were yet to be met, and one of these was that of a night nurse, which would involve an additional £60 a year. The Treasurer has given this sum for the first year, and this will be a comfortable post for a widow or elderly nurse. There was another department of the institution—that of supplying trained nurses for private nursing to persons who could afford to pay for them. There were now seven thoroughly trained nurses for any persons who required them, and an eighth was almost ready to be sent out. This part of the business of the institution was going on all right, as they had wiped off £39 of the £69 which they owed at the commencement of the year.

THOUSANDS OF POUNDS FOR THE NATIONAL PENSION FUND.—We have the pleasure to acknowledge the following sums towards the £14,000 now being raised to at once bring the Donation Bonus Fund up to £40,000. We hope to be able to acknowledge contributions amounting to £1,000 every week until the £40,000 are completed. Contributions of any amount may be sent to the Editor of The Hospital, or to the Manager, National Pension Fund for Nurses, 8, King Street, Cheapside, London, E.C.:

Ninth Thousand.

| | | | | | | | |
|---|---|---|---|---|---|---|---|
| Mr. E. Rawlings ... | £200 | 0 | 0 | Mr. G. E. Foster ... | £25 | 0 | 0 |
| Mr. Robert Gordon ... | 100 | 0 | 0 | Mr. E. B. Foster ... | 25 | 0 | 0 |
| Ditto (annual)... | 2 | 2 | 0 | Mr. C. F. Foster ... | 25 | 0 | 0 |
| Mr. C. Morrison ... | 100 | 0 | 0 | Sir W. R. Farquhar ... | 25 | 0 | 0 |
| Messrs. Helbert, Wagg | 100 | 0 | 0 | Messrs. Herries, Farquhar, & Co. ... ... | 25 | 0 | 0 |
| Messrs. Ferguson & Clark ... ... ... | 100 | 0 | 0 | The Skinners' Company | 21 | 0 | 0 |
| Messrs. Freshfields & Williams ... ... | 100 | 0 | 0 | Mr. G. W. Medley ... | 10 | 10 | 0 |
| | | | | Messrs. J. P. Trew & Co. | 10 | 10 | 0 |
| Messrs. Paine, Son, & Pollock... ... ... | 52 | 10 | 0 | Mrs. Rylands ... ... | 10 | 0 | 0 |
| | | | | Mr. D. Macneill... ... | 10 | 0 | 0 |
| Merchant Taylors' Co.... | 31 | 10 | 0 | Miss Lucy E. Loveday... | 1 | 11 | 6 |
| Mr. P. Williams... ... | 25 | 0 | 0 | Mr. E. Stopford Jones... | 0 | 10 | 6 |

Amount already received **Nine Thousand Pounds.**

DISTRICT NURSING AT GLOUCESTER.—There are two district nurses at Gloucester working under a superintendent, and doing quietly a very good work. The applications to the superintendent to nurse cases numbered last year 368. The number of persons nursed was 255, and the number of visits paid 5,832. These numbers significantly showed a continuous demand for nursing services; moreover, that the poor have unabated confidence in the aid rendered them through the agency of skilled nursing. Testimony from patients, their friends, medical men, clergy, district visitors, poor law guardians, and others is not wanting, and the Bishop of the diocese spoke most encouragingly of the society at the late annual meeting.

CHEERING THE PATIENTS.—A small entertainment, got up in a simple manner by the nurses and those of the patients who were able to help to amuse the others, took place at the Heart and Paralysis Hospital, 32, Soho Square, on Monday evening, March 10th. The pleasant little "evening" was opened by Nurse Andrews with "No, Sir," followed by a few other songs. Then came the piece of the evening, "The Pied Piper of Hamelin," a drametta in five characters. It was unanimously voted a success. Part II. was devoted to songs and small recitations, among the former "I Dreamt I Dwelt in Marble Halls" (Nurse Andrews), "Thy Voice is Near Me" (Nurse Nellie), and Mr. J. Warman sang very effectively "The Bells of Shandon." A touching little piece was delivered by Nurse Tilly, "The Fireman's Wedding." The enjoyment of the evening was greatly due to the kindness and unwearied efforts of the Matron and Nurses for the amusement of the patients.

EMIGRATION.—Last year a nurse wrote to us asking would we advise her to emigrate. We pointed out the pros and cons, and left her to decide for herself. Now comes a cheerful letter to us from the States, saying, "I arrived here on September 1, and carried round my certificates to the medical men, and the welcome I received was surprising, and far beyond my highest hope. I went to a case next day, and up to the present time have been busily employed, refusing more cases than I accepted. The Americans know well how to appreciate a good nurse." Our friend writes for The Hospital, and another correspondent in Italy asks for our paper. It is a great pleasure to us to think we are achieving such a wide circulation in other countries, and we always welcome communications from our correspondents abroad. Those nurses who emigrate seem to succeed when they have spirit and enterprise, and are able to work without the routine and appliances of hospital life; but again other nurses, not so strong and cheerful, send us woeful accounts of their difficulties in the wilds.

DISPENSING.—We so often have queries respecting dispensing that we are glad to be able to give the following particulars of Warneford Hospital, Leamington, where Miss E. M. Swain is resident dispenser. Pupils are received to learn dispensing on the following terms: (1) Each pupil must be approved by the committee. (2) The usual course of tuition extends over six months, and comprises instruction in dispensing and the compounding of the officinal preparations of the pharmacopœia, with elementary chemistry and materia medica. (3) The hours of attendance are each morning (Sundays excepted), from ten to half-past twelve, and for a variable period each afternoon. (4) While in the hospital the pupils will be required to conform to the rules of the institution, and will be under the control of the house surgeon and dispenser. (5) The pupil will not be allowed to dispense any medicines except under the immediate supervision of the dispenser. (6) The fee for six months' tuition is ten guineas (payable in advance). Arrangements may be made with the dispenser for a longer or shorter period. Special terms for nurses.

### Nursing Lectures.

By Percy Lewis, M.D.

Lecture V.—CHIEF DANGERS OF CHEST CASES.

The special danger to look out for in heart cases is a fatal syncope or faint from patients suddenly getting up or exerting themselves. These are trying cases to nurse, as there are frequently excitable people, whom it is very difficult indeed to keep still. I have several times seen patients die suddenly in the wards from some exertion, or getting out of bed, although strict orders to the contrary have been given. Now and then this will happen, in spite of the utmost vigilance and care on the part of the nurse, though, generally speaking, the more vigilant a nurse is, and the more she has her patients in hand, the less likely are these undesirable occurrences to take place. You will find it very difficult to get these patients comfortable in bed. Different cases want to be propped up at different degrees, and are constantly slipping down and requiring to be raised again. In these diseases it is important that the props should be so arranged as to support the patient chiefly *below* his chest, otherwise his breathing will be made more difficult.

To raise patients in bed, two nurses are in most cases required. One stands on either side, and putting one hand under his buttock, or upper part of his thigh, and the other under his back in the scapular region, both lift together. It is very distressing to see a nurse vainly endeavouring to drag up a heavy dropsical patient by the shoulder, to say nothing of the discomfort to which it puts the patient. I have known nurses attend very carefully to orders that these patients must be kept lying down, but who yet think it quite right to sit them up in a chair for several minutes every morning while they make their beds. This is a most dangerous proceeding. Patients with heart disease are not generally nice cases to deal with; they are hardly ever satisfied, and are constantly making complaints. Often you will find it very hard to keep your temper with them, and attend to them with that cheerful, even manner which a nurse should always strive to maintain.

Dyspnœa is almost as common a symptom of heart disease as it is of lung disease. It depends on the inability of the weakened heart with incompetent valves to get sufficient blood to the lungs to be aerated. You may relieve this form of dyspnœa to a certain extent by simply fanning their faces. All cases of bad dyspnœa, on whatever cause depending, should be saved the exertion of talking as much as possible. Never bother them with unnecessary questions, but get into the way of finding out their wants without asking them, and of anticipating them. It is a difficult power to acquire, but "practice makes perfect."

Restlessness is a symptom common to many diseases, depending, however, very often on slight and removable causes, such as too many bedclothes. A patient who has been tossing about half the night will frequently drop off to sleep on removing one of his blankets. Cold feet is another cause which should be met by hot water bottles. In giving these it is not sufficient to simply put them in the bed; they must be wrapped up in flannel or a blanket and put close to the patients' feet, without of course burning them. Perhaps, however, the cause most likely to be forgotten is a distended bladder. This should always be borne in mind, hence again you see the importance of observing systematically.

In case of a sudden faint or syncope, what should you do? In hospital the patient should be left lying down, the house-surgeon sent for, and while this is being done stimulants should be got ready. No stimulants should ever be administered in hospitals where a doctor is always available without orders. Supposing, however, that you are a long way away from medical aid, what should you do? It is not every case of syncope which requires stimulants, for internal bleeding gives rise to syncope, and internal bleeding may take place in a variety of diseases, as heart disease, ulcer of stomach, aneurism, etc. Syncope is Nature's way of arresting the bleeding; to give stimulants in these cases is only to renew the hæmorrhage. If, then, you suspect that the faint is due to hæmorrhage *don't give stimulants.* In any case withhold them as long as possible. You will nearly always be safe in laying the patient down, loosening the things round his neck, fanning his face, putting hot water bottles to his feet, smelling salts to his nose, and in opening the windows. If when you pour a little liquid into his mouth he makes no effort at swallowing, but only a gurgling sound, do not persist, for it will in all probability only go into his lungs. Should you have been warned that fainting was to be expected you will of course have all the restoratives within reach.

Cases of pleurisy with large effusions have been known to die of syncope from sudden exertion just as heart cases do. They may, in fact, be regarded as heart cases, for the fluid is pressing on the heart and impeding its action; until tapped they should be kept very quiet. Aneurism cases also should be treated as heart cases, the danger of exertion being if anything greater. These cases are treated in various ways, either by operation or medically. Tufnell's treatment consists in keeping the patient at absolute rest in bed for several months, avoiding all exertion of any kind, and with this view the regular administration of laxatives. Also in cutting down the diet to the least quantity required to maintain life. Tufnell's treatment is spoken of as the "starvation" treatment.

From interference with the circulation of the brain, cases of heart disease are apt to wander, or even have violent delirium. Cases, too, occur where they become permanently insane—the insanity of heart disease. Other organs may get affected for the same reason; patients may go blind, have hæmorrhage from the lungs, stomach, or kidney, have jaundice, dyspepsia, dropsy, &c. You will find that the class of cases we are discussing will get fits of depression, fancying they are going to die, and perhaps even refusing food. A nurse can do a great deal for these patients if she will take the trouble, by cheering them up, amusing them, and doing anything which will occupy their thoughts, and so prevent them thinking of themselves. Lots of cases of heart disease which you see in hospital improve sufficiently to go on with their work for several years, especially if they are able to lay up occasionally for a week or so to rest, a fact which gives you sufficient grounds for raising their hopes. Ventilation will form the subject of a future lecture. In the meantime remember that a ward should be maintained at an even temperature, for chest cases about 60 deg. Fahr.; draughts should be avoided, but the air kept pure.

There is one occasion when nurses are apt to get flurried, and that is at the visit of the physician. Everything should be done quietly and in an orderly and business-like manner. If he is in the habit of sitting down at a bed, a chair should be provided at each. Poultices should be so timed that they require removing about the time of his visit. As he comes to each bed he should find the patients undressed ready for him, but well covered by the bed clothes; thus he is not kept waiting. It is easy to find out from the house-surgeon in the morning what cases he is likely to want to examine. When a physician says he is going to examine a chest, he wants to see the whole of it. Now, from what I have told you of the extent to which the lungs reach upwards and downwards in the chest, it will be quite obvious to you that it is of no use to either open the night shirt a button or two at the top, or to pull it up from below. Only the part which he wishes to examine, such as chest or abdomen, should be uncovered at a time; still, do not go to the other extreme of pushing blankets, &c., unduly in his way. All windows near the case being examined must be closed. The most perfect silence must be maintained throughout the ward. No one must be allowed to walk about or even speak, for the sounds which we have to listen for are very feeble, and easily obscured by chattering voices or squeaky boots. Should it be a cold day, hot water must be ready in the basin on the table, so that the physician may warm his hands before applying them to the chest. A nurse should have ready a measuring tape and a blue aniline pencil, with a little water in a medicine glass to moisten it, in order that the physician may mark out various things on the chest. On no account should these marks be washed out without permission.

### Everybody's Opinion.

[*Correspondence on all subjects is invited, but we cannot in any way be responsible for the opinions expressed by our correspondents. No communications can be entertained if the name and address of the correspondent is not given, or unless one side of the paper only be*

### THE DUTIES OF A UNION NURSE.

VERITAS writes : I lately took a post as charge nurse of an infirm ward in a Union; after I had been elected I was informed by the matron, that in addition to my duty in the ward, I should be expected to take and fetch children to and from school; the Union having no school of its own sent them to various district schools. I did not at all like the idea, but made up my mind to give it a trial. My first journey was on a bitterly cold day, and took me from my ward for five hours. I had to fetch two boys back to the Union. The same evening I wrote to the master, telling him I found such work very objectionable and should like to be relieved of it, and that failing this, only one alternative remained. On the following morning the master came to the ward, and in the presence of my patients, informed me that no alteration would me made in an arrangement which had acted admirably for four years. We had rather high words, when he requested me to produce my rules, but I had never had any rules given me. Later on in the day, he gave me the rules, the fifth clause of which was as follows, "To perform such other duties as the Guardians, Master, or Matron may require." I should like to know if any of your readers have ever met with a similar experience, either in escorting pauper children to school, or having such an indefinite and unmodified rule laid down as the one I mention. I am given to understand that a board of guardians have power to create and enforce any fresh rules they deem fit. It appears to me arbitrary. Many of my patients are very aged and infirm, and during my absence are left in care of an inmate, acting as wardsman. Another duty I was called upon to perform was to be in attendance in the master's kitchen, on such days as the guardians take luncheon at the house, ostensibly to see that the inmates who wait upon them take in the courses correctly, but in reality, it is to see that they eat nothing which comes from the table of these guardians of the poor, who have a luncheon of three or four courses, with beer or whiskey and soda, for which they pay the large amount of one shilling per head. Being an experienced nurse, I have declined the honour of retaining such a unique appointment.

### IDIOT CASES.—WHO WILL HELP?

IN reference to the paragraph headed as above in our issue of the 1st inst. regarding the case of an idiot child, and asking for advice and assistance thereon, a correspondent, one of the founders of the Earlswood Asylum in 1847, says: "The case would doubtless be eligible for that asylum, and, if elected, would receive gratuitously five years' care and training, but it is difficult to obtain enough votes to secure the election, and considerable delay must occur before admission can be obtained. . . . . If the parents reside within the sphere of the Metropolitan Asylums Board, admission to their excellent asylum at Darenth would probably be obtainable through the board of guardians, payment to be made according to the means of the parents. Or in the same way two other asylums might be available, the Eastern Counties Asylum, Colchester, or the Western Counties Idiot Asylum, Starcross, Exeter. The rate of payment in the former would be 10s. 6d. per week, and at the latter 10s. per week. Towards this sum a subvention of 4s. per week is allowed by the Government, if the case is placed in the asylum through the parish. At the Royal Albert Asylum for the seven Northern Counties, at Lancaster, cases are received without election at reduced rates, as may be decided by the committee, according to the circumstances of the friends, the lowest charge probably being £30 per annum. Cases are also received through the parish authorities, at the rate of payment charged at the Lunatic Asylum for Pauper Lunatics, and three guineas extra for clothing. A Government subvention of 4s. per week is obtained for each case by the guardians. Cases must belong to one of the seven Northern Counties to be eligible for admission at the reduced rate of payment, or through the parish authorities. The Midland Counties' Asylum is at Knowle, near Birmingham, and receives cases from the five Midland Counties. Their reduced payment is £27 per annum, and £5 for clothing. No pauper cases are admissible. It is a small asylum. There is a still smaller asylum at Coombe Down, near Bath, and the lowest rate of payment is about 12s. per week, clothing to be supplied by the friends. A new building for fifty cases will soon be erected. Cases must be under fifteen years of age. No Government subvention is allowed for cases at Darenth, which is carried on under the Metropolitan Asylums Act. Parents are not pauperised if they pay for their children to the parishes, through which admission is obtainable, at reduced rates of payment. There are permissive clauses in the new Lunacy Bill authorising county councils to provide separate asylums for idiots. The County Councils of Lancashire and Essex have definitely proposed preliminary steps for the consideration of the subject. In Northamptonshire an idiot asylum has been erected upon the ground of the County Lunatic Asylum, at Berry Wood. It may be hoped that other county councils will speedily follow this excellent example." Of the suggestions given above, the one relating to Darenth seems the most feasible, and there appears to be a probability that this course will be tried first. We have to thank our correspondent for the trouble he has taken in furnishing us with these particulars.

### THE ZENANA MEDICAL COLLEGE.

MISS ROBINSON writes: At the present time, when the subject of female medical missions is engrossing the minds of Christian men and women of all denominations, who seem now to realise that this is the kind of service most adapted to meet the needs of India, China, &c., we desire to bring the following facts to the notice of your readers. The Zenana Medical College does not profess to send out missionaries, its aim being to prepare its students for this great work, and the report for the year will show that the object has been most successfully attained. The testimony of those who have seen the work done, both at home and abroad, by our students is strongly confirmatory. Miss Proctor, writing from Syria, says: "Few institutions can point to such a band of successful workers as Dr. Griffith can show from his college," and so impressed is she with the value of the instruction received here, that at her earnest request the committee have raised the necessary funds to give free training to two of her Syrian pupils, who, when trained, will return as medical missionaries to their own land. These will be the first Syrian women ever so trained, and Mr. R. C. L. Bevan has most kindly contributed £50 towards the free training of a third Syrian lady. The committee will thankfully acknowledge help from other friends who may feel disposed to follow Mr. Bevan's generous example. Miss Hewlett, of Amritsar, in her book on "Need of Healing," bears the following testimony, "I consider that Dr. Griffith's Zenana Medical College offers the best advantages for such training under the most favourable circumstances." This is a most valuable statement, because Miss Hewlett's two assistants were trained at the College—Miss Sharp, whose work at St. Katherine's Hospital, Amritsar, is well known to the writer from practical experience, and Miss Bose, the first Christian native lady trained in this country, who is in charge of the dispensary at Turan Turan. A third testimony is borne by Mr. W. S. Caine, M.P., who at the annual meeting in December said, "I may tell you that it is officially stated that one of the best dispensaries in the north-west provinces is under the charge of one of your former students. If, however, one thing be more important than another it is the branch of maternity, and in that your students pass well." The report may be had from the secretary, 58, St. George's Road, W.

### Death in our Ranks.

THE twenty-sixth annual report of the St. Luke's Free Hospital, Chicago, just received, contains the following notice of Miss Sarah B. Miles, for nineteen years the matron of that institution. We reproduce it because it affords pleasing testimony to the stability and standing of American institutions, and sets an example which might be far more generally followed than it is at present. We hold that devoted labourers in the field of charity should have their deeds recorded in the archives of the institutions which have benefited by their works and labour of love. The report says: "Whereas on God's day of rest, the 30th of December, 1888, Miss Sarah B. Miles passed from within the walls of St. Luke's Hospital into the eternal rest of Paradise, the trustees of the hospital desire to place upon record this minute, as a testimonial of their affection and respect for her memory, and of their appreciation of her long and devoted services to the hospital. During the nineteen years while she was its matron she fulfilled the duties of that position—which embraced many more labours and responsibilities than ordinarily appertain to such a position—with earnest fidelity and unwearied zeal and enthusiasm. Under her fostering care it was carried through the earlier days of its history with great wisdom and devotion, when faithfulness and prudence were so essential to its success. A woman of wonderful executive ability, careful in expenditure, watchful over every department of housekeeping, honourable and upright in her management, thoughtful and tender toward the patients, in harmony with the physicians, promoting interest among the patrons of the institution, untiring in all her efforts, she counted no sacrifice of self too dear for the welfare of the hospital whose interest lay so near her heart. A thorough Christian and conscientious churchwoman, she has left to us the legacy of a noble character, and an example of zeal and devotion to St. Luke's Hospital, which, we trust, will incite many to generous and self-sacrificing deeds for this blessed institution of Christian charity. Many, as they recall the life and services of our late-lamented matron, will rise up and call her blessed."

THE Islington Board of Guardians have put on record the long and faithful labours of the late mistress of the workhouse and infirmary. Mrs. Sustins literally died in harness, and only five hours before her death she was superintending the weekly changing of the bed linen, and only desisted at the earnest request of the doctor. There was no doubt that if she had laid up sooner she would have escaped the severe cold which had terminated in her death. By her decease the Board had lost a splendid officer, and a vote of sympathy with Mr. Sustins was passed.

### Presentation.

MISS EVANS has just been presented with a handsome timepiece and other gifts by the nurses and household of the Stockport Infirmary, on the occasion of her resigning the office of matron.

### Amusements and Relaxation.

### FOURTH QUARTERLY WORD COMPETITION

Commenced Jan. 4th, 1890, ends March 29th, 1890.

The word for dissection for this, the TWELFTH week of the quarter, being

"BISMARCK."

| Names. | Mar. 13. | Totals. | Names. | Mar. 13. | Totals. |
|---|---|---|---|---|---|
| Holland | 85 | 949 | Lauriston | 83 | 956 |
| Red Cape | — | 132 | B. E. T. | — | 453 |
| Nurse Marie | — | 499 | Jacko | — | 110 |
| Garfield | — | 243 | Lady Clara | — | 258 |
| Midget | 60 | 814 | Patience | 89 | 999 |
| E. M. S. | — | 149 | Ecila | 85 | 984 |
| Sister | — | 591 | Maude | — | 690 |
| Duchess | — | 585 | Esperance | 79 | 971 |
| Ellice | 91 | 959 | Ruby | — | 698 |
| Reldas | 91 | 999 | M. W. | 81 | 972 |
| Jenny Wren | 75 | 860 | Crystal Palace | — | 568 |
| Mopus | — | 122 | Jackanapes | 63 | 724 |
| Neptune | — | 118 | M. M. M. | — | 25 |
| Qu'appelle | 67 | 892 | A. F. | — | 318 |
| Whiteboe | 50 | 841 | E. S. | 49 | 49 |
| Nurse Clague | — | 40 | | | |

### Vacancies.

THE following vacancies are announced:

### Matrons.

Gateshead Children's Hospital; £36.

Royal Albert Edward Infirmary, Wigan; £60.

West Cornwall Infirmary; £40.

Rochdale Infirmary; £40.

### Assistant Superintendent.

Leeds General Infirmary; £50.

### Sister.

Stafford Infirmary.

### Nurses.

Aimee Nursing Home.
Bournemouth Nursing Institution; £25.
Bedfordshire Nursing Institution; £28.
Brompton Consumption Hospital; £25.
Burton Infirmary; £25.
Burton-on-Trent Institution; £25.
Bournemouth Victoria Home; £25.
Bedford General Infirmary (night); £20.
Blackheath and Richmond Nursing Institution.
Chester Deaconess House; £24.
Central London Sick Asylum; £20.
Cardiff Infirmary; £24.
Central London Throat and Ear Hospital; £14.
Christ's Hospital, Hertford (temp.).
City of London Chest Hospital; £20.
Coventry and Warwickshire Hospital (night); £20.
Devonport Albert Hospital; £25.
Exeter Hospital; £24.
Eastbourne Borough Sanatorium; £30.
Edinburgh Poorhouse Hospital; £25.
General Nursing Institute.
Glasgow Hillhead Home; £30.
Huddersfield Infirmary; £24.
Hull Nurses' Home.
Hanover Institute, W.; £25.
Her Majesty's Hospital; £25.
Holborn Union (night); £20.
Hospital for Incurables, Mauldeth; £30.
Johnson Hospital, Spalding; £20.
Jessop Hospital, Sheffield; £20.
Kendal Hospital; £28.
Kent Nursing Institution; £30.
Liverpool Eye Infirmary; £20.
Mr. Wilson's Institution.
Massachusetts; £40.
Northampton Nursing Institution; £25.
Notts Nursing Association.
North-Western Fever Hospital; £30.
Pendlebury Hospital; £20.
Royal Chest Hospital; £22.
St. Vanda's Home, Bromley.
Southport Nurses' Home.
Southport St. John's Institution.
St. Saviour's Infirmary; £20.
Stamford Infirmary; £25.
Shoreditch Infirmary; £20.
Smithston Hospital, Greenock; £25.
Swansea and S. Wales Nursing Institution; £25.
Tunbridge Wells Hospital; £25.
Tewkesbury Hospital (night).
Wolverhampton Victoria Institution.
West London Hospital; £28.
Westminster Ophthalmic Hospital; £20.
Yeovil Hospital; £14.

### District Nurses.

Benefit Nursing Association.
Burton-on-Trent; £25.
Great Bedwyn, Wilts; £25.
Haverfordwest.
Leeds District Home; £25.
Middlesbrough Nursing Association.
Norbiton; £70.
Queen Victoria Nurses' Institute (Scottish Branch).
Upper Tooting; £50.

### Probationers.

Burton-on-Trent Infirmary.
Guildford County Hospital.
Kings Lynn Hospital.
Notts Nursing Association.
Newport Infirmary.
Royal Maternity Charity.
Southport Massage Institution.
Workhouse Infirmary Nursing Association.
Wirral Children's Hospital.

### Notes and Queries.

### Queries.

(50) Will Sister Dora, who so kindly answered my query about boots for distict work, tell me the address of the head-quarters of the india-rubber company she mentions? And also say whether the boots with rubber soles are not too hot for summer wear? Would not the rubber soles cut out very rapidly on rough country roads in a very hilly district? I am obliged to have nails in my boots, like men, as it is.—*A Country District Nurse.*

(70) *Publishers' Address.*—Can anyone kindly give me the address of the publishers (Bailliere, Tindall, and Cox) where I could obtain "Nursing Questions and Answers," by J. W. Martin, M.D.?—*E. F.*

(71) *American Hospitals.*—Where can I obtain a list of American hospitals?—*Nurse Francis.*

### Answers.

(63) "E. M. W." had better write to the Brownlow Hill Infirmary, Liverpool, for rules. The training there, which is excellent, embraces both general nursery and midwifery. I believe nurses have to sign for three years.

(64) *Uniform Cloaks.*—These can be had from Wallis, High Holborn. —*Barts.*

(70) *Publishers' Address.*—20 and 21, King William Street, Strand, W.C.

*The Bonus Fund.*—We beg to inform Nurse Norris that participation in the Bonus Fund commences at the same time as the pension. The Bonus Fund will be valued and credited every five years.

*Musical Nurse.*—There is a portable instrument to be had from any music-sellers, but it only consists of one octave, and is useful only in keeping the fingers supple.

(66) In reply to "Nurse," I beg to say that I have found the most effectual remedy to be spirits of turpentine and carbolic lotion, 1 in 40 equal parts. The head and hair to be thoroughly saturated every day till cured. The hair should be combed with a fine tooth-comb about half-an-hour after the application of the lotion.

*St. John Ambulance Ass.*—We beg to inform "Fern" and others that they must apply to the Sec., St. John's Gate, Clerkenwell, for particulars of this association.

*Nurse Andrews.*—The Hertford Hospital, Paris.

*Sister Ford.*—We regret we do not know the address of the Home mentioned, nor the terms.

